# Complex Minigene Library Vaccination for Discovery of Pre-Erythrocytic *Plasmodium* T Cell Antigens

**DOI:** 10.1371/journal.pone.0153449

**Published:** 2016-04-12

**Authors:** Brad C. Stone, Arnold Kas, Zachary P. Billman, Deborah H. Fuller, James T. Fuller, Jay Shendure, Sean C. Murphy

**Affiliations:** 1 Department of Laboratory Medicine, University of Washington, Seattle, Washington, United States of America; 2 Center for Emerging and Re-emerging Infectious Diseases, University of Washington, Seattle, Washington, United States of America; 3 Department of Microbiology, University of Washington, Seattle, Washington, United States of America; 4 Department of Genome Sciences, University of Washington, Seattle, Washington, United States of America; 5 Seattle Malaria Clinical Trials Center, Fred Hutchinson Cancer Research Center, Seattle, Washington, United States of America; 6 Human Challenge Center, Center for Infectious Disease Research, Seattle, Washington, United States of America; Institut de Recherche pour le Développement, FRANCE

## Abstract

Development of a subunit vaccine targeting liver-stage *Plasmodium* parasites requires the identification of antigens capable of inducing protective T cell responses. However, traditional methods of antigen identification are incapable of evaluating T cell responses against large numbers of proteins expressed by these parasites. This bottleneck has limited development of subunit vaccines against *Plasmodium* and other complex intracellular pathogens. To address this bottleneck, we are developing a synthetic minigene technology for multi-antigen DNA vaccines. In an initial test of this approach, pools of long (150 bp) antigen-encoding oligonucleotides were synthesized and recombined into vectors by ligation-independent cloning to produce two DNA minigene library vaccines. Each vaccine encoded peptides derived from 36 (vaccine 1) and 53 (vaccine 2) secreted or transmembrane pre-erythrocytic *P*. *yoelii* proteins. BALB/cj mice were vaccinated three times with a single vaccine by biolistic particle delivery (gene gun) and screened for interferon-γ-producing T cell responses by ELISPOT. Library vaccination induced responses against four novel antigens. Naïve mice exposed to radiation-attenuated sporozoites mounted a response against only one of the four novel targets (PyMDH, malate dehydrogenase). The response to PyMDH could not be recalled by additional homologous sporozoite immunizations but could be partially recalled by heterologous cross-species sporozoite exposure. Vaccination against the dominant PyMDH epitope by DNA priming and recombinant *Listeria* boosting did not protect against sporozoite challenge. Improvements in library design and delivery, combined with methods promoting an increase in screening sensitivity, may enable complex minigene screening to serve as a high-throughput system for discovery of novel T cell antigens.

## Introduction

*Plasmodium* parasites cause malaria, a mosquito-borne disease responsible for hundreds of thousands of deaths and hundreds of millions of clinical cases annually [1]. After transmission from feeding Anopheline mosquitoes, injected sporozoite-stage parasites travel through the skin and eventually make their way to the liver where they initiate an asymptomatic hepatocyte infection that culminates days later in release of erythrocyte-stage merozoites. The merozoites initiate a cyclical infection of erythrocytes that leads to all the clinical manifestations of malaria, including fevers and chills and sometimes progressing to severe anemia, coma and death. The single greatest advance in the fight against malaria would be the production of a safe and effective vaccine that induces complete protection against infection with *Plasmodium* sporozoites. To achieve this goal, a major focus has been placed on vaccines targeting the pre-erythrocytic stages of development (the transmitted sporozoite stage and the subsequent liver stage) [2]. Experimental vaccination of humans and mice with attenuated sporozoites protects against challenge with wild-type sporozoites [3–8]. Antibodies can block hepatocyte invasion [9], while CD8^+^ cytotoxic T cells recognize parasite-infected hepatocytes [10–12] and can provide sterile protection in several mouse models [13]. Furthermore, CD8^+^ T cells play a role in protection in primates [14] and likely in humans as well [15] and are induced in humans experimentally immunized with attenuated sporozoites [16, 17]. Parasite-specific CD8^+^ T cells may kill infected cells by one or more proposed mechanisms (reviewed in [18]). Thus, inclusion of CD8^+^ T cell target antigens is likely to be critical for any sterile protective malaria vaccine.

There are two basic approaches to pre-erythrocytic malaria vaccine development–manufacture of an attenuated ‘whole organism’ vaccine or identification and manufacture of a single subunit antigen or set of antigens that can provide complete protection [19, 20]. However, the extremely large number of genes expressed by *Plasmodium* has thus far prevented the efficient identification of broadly protective antigens suitable either for inclusion within a subunit vaccine or for targeting by transgenic parasite vaccines with improved protective efficacy.

For subunit vaccines, there are two sequential phases of development: antigen discovery and formulation testing. Antigen discovery typically involves screening of immune cells from pathogen-exposed subjects for responses against small numbers of laboriously cloned, pathogen-derived gene products or predicted peptide epitopes. Antigen discovery is then followed by an equally laborious process of production and evaluation of experimental vaccines targeting the newly discovered antigenic proteins–many vaccines fail here because the antigens discovered simply do not induce protective responses. As the number of proteins increases, each step requires considerable investment of time and money, with no guarantee that the antigens discovered will ultimately confer protection when formulated as a vaccine.

*Plasmodium* species each encode ~5,300 proteins but only a few pre-erythrocytic *Plasmodium* proteins (i.e., CSP, TRAP, LSA1, Exp1/Hep17, CelTOS, L3, Pf16, STARP) have been studied as T cell antigens [21–31]. Amongst those tested in humans (CSP, LSA-1, Exp-1, TRAP, CelTOS), most have not reliably induced complete protection (reviewed in [32]), although CSP protected 85% of subjects in one study [33]. Because it is nearly impossible to systematically study all potential T cell antigens using conventional methods, a number of higher throughput approaches have been applied to malaria T cell antigen discovery. Synthetic *Plasmodium* peptides [34–37] or antigen presenting cells (APCs) transfected with *Plasmodium* protein-expressing plasmids [38] have all been used to screen for T cell interferon-γ (IFNγ) responses *in vitro*, but such approaches are limited by the cost of large peptide libraries and the complexity of cloning A/T-rich Plasmodial genes, respectively. Recently, a peptide screening approach identified multiple new antigens targeted by T cell responses from RAS-immunized humans [37]—protection afforded by these antigens is not yet known. We previously utilized APCs transfected with minigene libraries encoding long *Plasmodium* peptides in an effort to capture a larger portion of the proteome using synthetic biology techniques [26].

New methods that accelerate discovery of vaccine subunits for pathogens with thousands of genes would be a major step forward in the fight against *Plasmodium* and other complex intracellular pathogens. DNA vaccination and screening is ideally suited for such higher-complexity evaluation of vaccine candidates. In malaria, DNA vaccination against *P*. *yoelii* CSP achieves CD8^+^ T cell-dependent protection in the rodent model [39]. In addition, DNA vaccination of humans against *P*. *falciparum* CSP can induce CD8^+^ T cell responses [40]. Finally, small numbers of *Plasmodium* protein-coding genes have been shown to be immunogenic when used in a four-gene DNA vaccine in mice or non-human primates [41, 42]. A five-gene DNA vaccine administered to humans was also able to induce CD8^+^ T cell responses that could be further boosted by exposure to sporozoites [43]. Even though there was no evidence of protection in this human study, the study showed that a DNA prime / *Plasmodium* boost protocol can increase responses. Studies from outside the malaria field further indicate that complex DNA vaccines delivered by biolistic (gene gun) delivery can be physically segregated onto distinct gold beads and that this serves to maintain diversity of both antibody and T cell responses [44–46].

We previously used a high-throughput synthetic minigene technology for *in vitro* detection of murine CD8^+^ T cells primed with live *Plasmodium* parasites *in vivo* [26]. This work led us to hypothesize that these same minigenes could be used to both initiate specific immune responses *in vivo* as a DNA-based vaccine, and to subsequently screen DNA vaccine-induced T cell responses to identify antigenic targets. This approach potentially combines the antigenic complexity achieved by whole organism vaccines with the feasibility of multi-subunit vaccination. Using a microarray-based oligonucleotide synthesis technology, we rapidly produced two complex minigene vaccines, each encoding over >1,000 peptides derived from 36 (vaccine 1) and 53 (vaccine 2) liver-stage *P*. *yoelii* proteins. Targets were a set of pre-erythrocytic proteins containing signal peptides and/or transmembrane domains. Putatively secreted or transmembrane proteins were selected based on their potential to cross both the parasite membrane and the parasitophorous vacuolar membrane to enter the hepatocyte MHC class I pathway. The assumption that such parasite proteins may be exported into the hepatocyte is based on the observation that exported erythrocyte-stage PEXEL/HT domain-containing proteins also contain a signal peptide or a transmembrane domain [47, 48].

Following vaccine production, mice were repeatedly gene gun vaccinated with the minigene libraries followed, in some mice, by sporozoite exposures. T cell IFNγ responses were evaluated and several novel responses were identified including a strong response to *P*. *yoelii* malate dehydrogenase (PyMDH), which was further characterized and found to bear similarities to another recently described *Plasmodium* liver-stage T cell response.

This preliminary study indicates that T cell responses against genuine Plasmodium antigens can be induced and detected using highly complex DNA minigene vaccines. As described in the discussion, additional improvements designed to increase the breadth and magnitude of responses, coupled with improved screening sensitivity may allow minigene vaccination/screening technology to be used for high-throughput identification of protective T cell antigens.

## Materials and Methods

### Vaccine design

Eighty-nine liver-stage *P*. *yoelii* 17XNL proteins predicted to contain a signal peptide and/or have transmembrane domains were selected using filters built into PlasmoDB. Coding sequences were downloaded and broken into sequential 33 codon segments overlapping by 14 codons. In regions of variability, all permutations of closely-spaced variations were included as alternate minigenes. A pool-specific primer unique to groups of 10 minigenes followed by a start codon were appended onto the 5’ end of each segment, while a common primer sequence was appended onto the 3’ end. The reverse compliment of each 150 bp minigene template was ordered as a single oligo-pool synthesis (CustomArrays, Inc., Bothell, WA). Gene identifiers and product descriptions from PlasmoDB.org as well as pool-specific primers, minigene sequences and encoded peptides are listed in **S1 Table**.

### Vaccine assembly

Each pool of 10 minigenes was amplified using individual pool-specific primers plus the common primer. Pool-specific primers included a T7 promoter sequence on the 5’ end. Equivalent amounts of each pool were then combined and subjected to dial-out error correction as described [49]. Briefly, random tags were added by amplifying the combined library using stepout primers hybridizing to the T7 and common primer sequences. The tagged library was sequenced using base paired-end reads on an Illumina miSeq by a commercial vendor. Reads were aligned with the original library design and dialout primer pairs flanking accurate minigenes were selected. Dialout primer pairs for each minigene were ordered from IDT, Inc. (Coralville, IA). Primers could not be adequately designed for some minigenes and such minigenes were omitted from the final library post-dial out. Error-corrected minigenes were amplified using the selected dial-out primers. Each minigene was individually recombined as an amino-terminal fusion with the mouse LC3 coding sequence in a modified pNGVL3 vector using SLiCE ligation-independent cloning [50]. Recombined plasmids were transformed into DH10G *E*. *coli* hosts by electroporation using an AMAXA 96-well shuttle device (Lonza, Walkersville, MD) and cultured individually in 1.2 mL LB broth in deep-well 96-well plates. Groups of 10 cultures were pooled, centrifuged and frozen until purification. Bacterial pellets were processed using a 96-Plus Endotoxin-free kit (Qiagen, Valencia, CA) according to manufacturer instructions. Typical yields were between 100 and 300 ng/μL for each pool.

### Loading of gene gun cartridges

Nine μL of each plasmid pool, each containing 10 plasmids, were combined with 1 μL of 200 ng LT adjuvant plasmid [51] in 96-well V-bottom plates. Each well was diluted with 10 μL 50 mM spermidine and 1 mg of 1 μm gold beads (Inbios Gold, Hurstbridge, Australia). Plates were agitated on a horizontal shaker while 10 μL 10% CaCl_2_ was added to each well. DNA-coated gold particles were centrifuged and washed thrice with 100% ethanol. Gold particles were suspended in 10 μL 100% ethanol with 50 μg/mL polyvinylpyrrolidone. One-quarter of all pools in a vaccine (the equivalent of twelve 40-kD proteins) were combined and loaded into Tefzel cartridges using a custom-made tube turner. Gene gun cartridge tubes were dried, sliced and stored desiccated at 4°C until use.

### Mice

All animal studies were approved by the University of Washington Institutional Animal Care and Use Committee (protocol 4317–01). BALB/cj mice were obtained from Jackson Laboratories (Bar Harbor, ME) and housed in approved facilities at the University of Washington. Thy1.1^+^ BALB/c mice were bred at the University of Washington from a pair originally obtained from Jackson Laboratories. Humane sacrifice was performed by flow-metered carbon dioxide overdose.

### Gene gun vaccinations

Female Balb/cj (6–8 wk old) mice were shaved on the abdomen and administered the DNA vaccine corresponding to 0.5–1.0 μg total DNA using a PowderJect XR1 research device [52]. On Days 0, 21 and 49, each mouse received four separate cartridges, one cartridge corresponding to each quarter of the vaccine. At later time points, vaccinated mice were humanely sacrificed and splenocytes harvested and pooled for screening as described later.

### Generation of screening templates

One μL of each vaccine pool was diluted 1:100 and further amplified using Rolling Circle amplification using a TempliPhi kit (GE BioSciences, Pittsburgh, PA). Rolling circle-amplified DNA from each minigene pool was used as a template to amplify short linear expression cassettes from the CMV promoter through the human beta-globin 3’UTR/polyA sequence. PCR reactions utilized 20 ng template combined with a CMV promoter-specific primer and a 3’ human beta-globin UTR antisense primer and Phusion polymerase. Cycling conditions were 35 cycles of 98°C (15 sec), 55°C (15 sec) and 72°C (2 min) each. Products were purified using 96-well filter plates (Qiagen, Valencia, CA) and resuspended in 20 μL 10 mM Tris (pH 7.8).

### Minigene library ELISPOT screening of sensitized splenocytes

Ready-Set-Go mouse IFNγ ELISPOT kits (eBioscience, San Diego, CA) were used as previously described [26]. Briefly, 2 μL of PCR-amplified expression cassettes from each minigene pool in each vaccine were transfected into 1x10^6^ freshly-dividing P815 cells using the RAW264.7 program of an AMAXA 96-well shuttle. Splenocytes (1x10^6^/well) were added as responders, and plates were incubated overnight. ConA was included as positive control and mock-transfected P815 cells were used as negative controls. ELISPOT plates were developed according to the manufacturer’s instructions and scanned and analyzed using an ImmunoSPOT counter (Cellular Technology Limited, Shaker Heights, OH). Transfection efficiency was also controlled by checking GFP expression and viability by flow cytometry of separate wells of P815 cells transfected on each plate of wells.

### Sporozoite immunizations

Where indicated, BALB/cj mice were singly or doubly immunized with 1x10^4^ wild-type *P*. *yoelii* sporozoites and were then administered azithromycin i.p. (0.8 mg/d) on Days 1–3 post-immunization. In other experiments, mice were immunized once or twice with combinations of PyRAS and/or genetically-attenuated Pyfabb/f- sporozoites [53] as described [26]. Sporozoites were obtained from the Center for Mosquito Production and Malaria Infection Research (CeMPMIR, Center for Infectious Disease Research, Seattle, WA).

### MHC binding assay

RMA/S cells expressing H2-K^d^ were used to test MHC binding as described [26, 54].

### *In vivo* cellular cytotoxicity assay

The *in vivo* killing assay was performed to measure cellular cytotoxicity as previously reported [26] although three populations of target cells were differentially labeled here with Cell Proliferation Dye eFluor® 670 (eBioscience) at 5 μM (PyMDH), 0.75 μM (PyCSP) and 0.1 μM (mock).

### Immunization-challenge experiments

To generate DNA and Listeria-based vaccines for single antigen immunization, minigene-encoded antigens were isolated and further cloned into the delivery vectors. Briefly, minigene expression constructs encoding the dominant PyCSP epitope SYVPSAEQI and the dominant PyMDH epitope SYQKSINNI were cloned separately into the pNGVL3.LC3 vector for use as DNA vaccines. Recombinant PyCSP-expressing *actA-/inlB- L*. *monocytogenes* was a kind gift from Aduro Biotech. PyMDH-expressing *actA- L*. *monocytogenes* was constructed by cloning a minigene encoding the PyMDH epitope into the pPL2-N4 vector. The resulting pPL2-N4.MDH plasmid was transformed into SM10 hosts and conjugated into *actA-* Lm10403S. Using these materials, a gene gun DNA prime/recombinant *Listeria* boost protocol was used to vaccinate mice against single antigens prior to sporozoite challenge. For each arm of the experiment, mice were primed against either PyCSP or PyMDH using two gene gun cartridges on Days 0 and 2. Three weeks later, animals were boosted i.v. with 5x10^6^ CFU recombinant *Lm* expressing the cognate antigen and treated with 2 mg/mL ampicillin in the drinking water for 3 days thereafter. Animals were challenged three weeks later with 1x10^4^ wild-type *P*. *yoelii* sporozoites i.v. Forty-four hr later, animals were humanely sacrificed and livers perfused with PBS. Livers were harvested and emulsified in a bead beater with NucliSens lysis buffer (bioMérieux, Durham, NC) to preserve RNA. Total nucleic acid was extracted using a NucliSens EasyMag (bioMérieux) and *Plasmodium* 18S rRNA and murine GAPDH were measured by RT-PCR as described [26]. Liver burden is reported as log_10_ changes between mice for *Plasmodium* 18S rRNA copy number normalized to mouse GAPDH. At the time of humane sacrifice, spleens were also harvested for ELISPOT analysis as a measure of T cell frequency at the time of challenge (no T cell expansion during 0–44 hr post-challenge).

## Results

### Synthetic biology methods enable rapid production of multi-protein synthetic minigene libraries suitable for T cell vaccination and subsequent screening

Two minigene libraries encoding peptides from 36 (Vaccine 1) or 53 (Vaccine 2) *P*. *yoelii* proteins each were synthesized (**S1 Table**). Error-correction including Dial-out tagging, sequencing, analysis and re-amplification was completed in 4 weeks. The libraries were designed to contain the complete peptide compliment of all included proteins, but after error correction by dial-out PCR, a fraction of minigenes could not be recovered as error-free sequences. Vaccine 1 contained 91.9% of the intended proteome (95%CI: 87.4–96.4%) and Vaccine 2 contained 91.1% (95%CI: 87.0–95.3%) as indicated in **S1 Table**. Plasmid insertion and plasmid culture/isolation were accomplished in three weeks. Spot-checking individual cultures by sequencing showed that >90% of clones encoded the intended sequence. The proteins included in the libraries are listed in **S1 Table**. Minigenes were adjuvanted with the LT-encoding plasmid [51] and loaded onto individual aliquots of gold particles for biolistic delivery.

### Library vaccination and minigene screening enables identification of library vaccine-induced IFNγ-producing T cell responses

**Fig 1:**
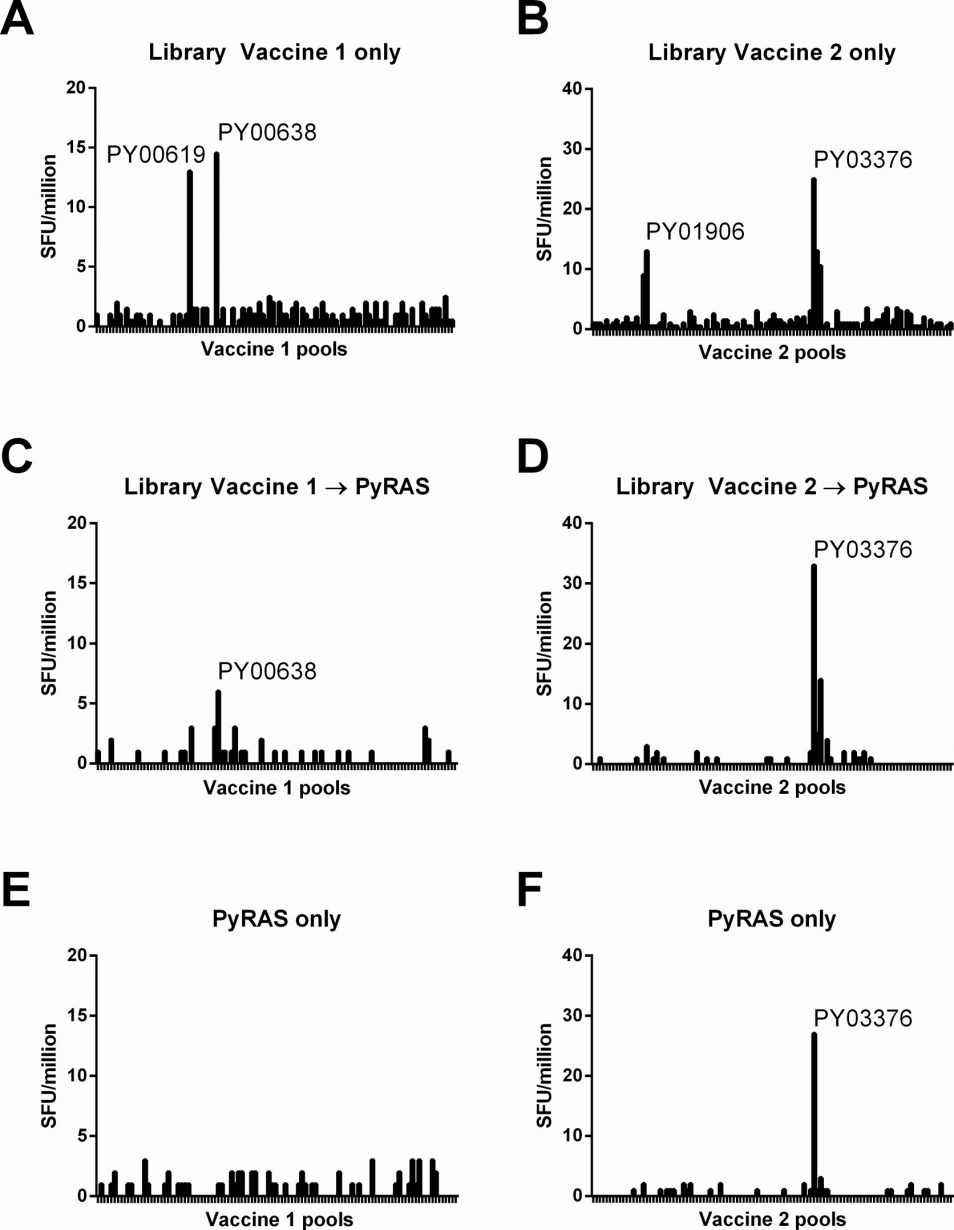
**ELISPOT IFNγ responses in mice immunized with minigene library vaccines alone (A-B), library vaccines followed by sporozoites (C-D) or sporozoites alone (E-F) identify novel responses.** ELISPOT results for Vaccine 1 (A,C,E) and Vaccine 2 (B,D,F), as indicated. Responses to Library vaccination alone (A-B), Library vaccine plus Sporozoites (C-D) or Sporozoites only (E-F). Minigene pools are indicated along the x-axis (Vaccine 1 = 103 pools; Vaccine 2 = 105 pools) with the y-axis indicating SFU/million splenocytes.

BALB/cj mice (n = 5/group) were vaccinated with Vaccine 1 or Vaccine 2 on Days 0, 21 and 49 using a PowderJect research device. Aside from mild erythema at the gene gun vaccination site for 1–2 days post-vaccination, the mice tolerated the DNA vaccination without any obvious signs of distress. IFNγ responses were evaluated usingpooled splenocytes harvested six days after the final gene gun vaccination. To evaluate the >100 minigene pools per vaccine, duplicate wells of target cells (P815) were each transfected with PCR-amplified expression cassettes corresponding to each minigene pool in a vaccine (10 minigenes/pool). The transfected target cells (1x10^6^/well) were combined with 1x10^6^ splenocytes from library-vaccinated mice in overnight IFNγ ELISPOT assays as previously reported [26]. Compared to previous minigene-transfected ELISPOT screens of sporozoite-immunized mice [26], background production of IFNγ in these experiments on DNA minigene library-vaccinated mice was extremely low (<3 SFU/million splenocytes). Such low background signal is critical for detection of responses in the setting of multi-protein vaccination. Two antigens in each vaccine induced responses significantly above baseline (Fig 1A and 1B). Each of the antigenic pools contained minigenes from a single protein such that the initial screen identified responses to the products of genes PY00619 and PY00638 in Vaccine 1 and PY01906 and PY03376 in Vaccine 2.

In Vaccine 2, the known epitope from the circumsporozoite protein (CSP, pool 55) did not yield detectable responses. There are several possible explanations for this result. First, a particular minigene may not express in an optimal manner for several reasons including failure to clone, presence of a PCR-induced mutation, inclusion of a suboptimal 5’ UTR which is dictated by the pool-specific primer, efficient shunting into the autophagosome via the LC3 tag [55], and/or poor representation relative to other minigenes in that pool. Another possible explanation is that ELISPOT screening using minigene-transfected P815 cells as APCs is less sensitive than peptide ELISPOTs since the minigene-transfected APCs express lower quantities of antigen, typically yielding spots of smaller diameter and lower intensity. To evaluate these possibilities, we subcloned the CSP epitope-containing minigene from Vaccine 2 using minigene specific primers and verified its presence and primary sequence in the library (data not shown). In a mixed minigene vaccination experiment using immunization with a single pool consisting of 20 linear minigenes with and without the *library-derived* LC3-tagged CSP minigene, we observed relatively low frequency responses (20–30 spots/million splenocytes) using CSP peptide as a recall antigen. When the CSP epitope was tested as a single minigene vaccine with a ubiquitin tag, this minigene could elicit strong CSP responses in IFNγ ELISPOTs (500–1000 spots/million splenocytes). Thus, it appears that the minigene encoding the major CSP epitope as a fusion to LC3 was functional but was unable to induce a response when formulated to at extremely high complexities. In addition, since we were unable to detect CSP responses in these experiments, we do not regard the lack of response against any library-encoded peptide as evidence that the target is not immunogenic or protective. Optimized delivery and increased dose are likely to induce responses against a larger percentage of the proteins targeted in these vaccines.

### A subset of library vaccine-induced responses are recalled by immunization with irradiated *P*. *yoelii* sporozoites but only one such response is primed by sporozoites alone

A subset of the minigene library vaccinated animals were administered 2x10^4^ irradiated *P*. *yoelii* sporozoites (PyRAS) 16 days after the final gene gun vaccination and splenocytes were harvested 7 days after RAS exposure. Minigene-transfected P815 ELISPOT screening was again conducted to identify vaccine-primed, RAS-recalled responses. Recall responses were observed against pools encoding PY00619, PY00638 and PY03376 whereas responses against the PY01906 pool were undetectable (Fig 1C and 1D). To determine if RAS alone could induce these responses, completely naïve BALB/cj mice were administered 2x10^4^ PyRAS and evaluated by minigene-transfected P815 ELISPOT one week later. In mice exposed only to PyRAS, the only detectable response was to the pool for PY03376, which encodes *P*. *yoelii* malate dehydrogenase (PyMDH) (Fig 1E and 1F).

### Identification of a Class I epitope in *P*. *yoelii* malate dehydrogenase

**Fig 2:**
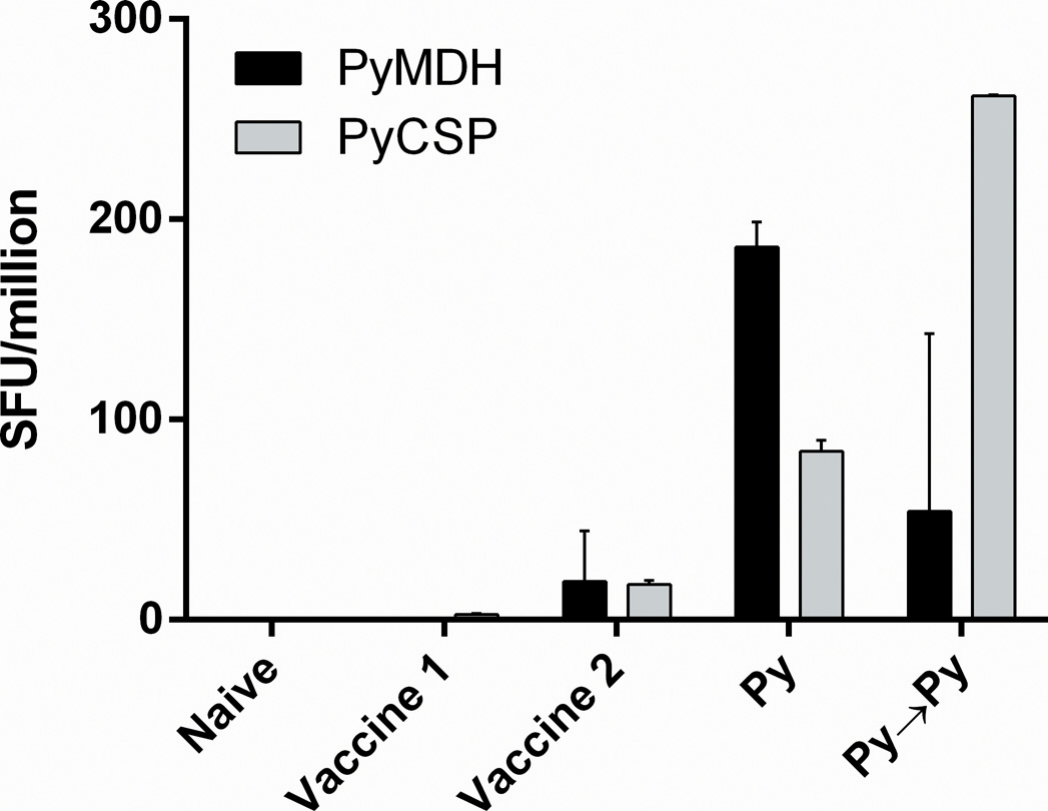
Identification of SYQKSINNI as a dominant natural epitope in *P*. *yoelii* malate dehydrogenase. Cryopreserved splenocytes were obtained from library-vaccinated mice (Vaccine 1 or Vaccine 2) or from mice singly (Py) or doubly (Py→Py) with doses of 1x10^4^ wild-type Py sporozoites administered with azithromycin (0.8 mg/d) on Days 1–3 post-immunization); for Py→Py, immunizations were spaced 3 wks apart. In all situations, splenocytes were harvested 6 d after the final immunization. Cells were thawed and tested by IFNγ ELISPOT against purified peptides corresponding to PyMDH (SYQKSINNI), PyCSP (SYVPSAEQI) and PyL3. Bars indicate mean and 95% confidence interval.

Three adjacent minigene pools from PyMDH were targeted in the primary screens. These pools included a total of 23 overlapping minigenes that were required to encode all permutations of four closely-spaced, non-synonymous sequence variants reported in the genome of *P*. *yoelii* strain 17XNL. It is unknown whether these reported variations are genuine or are the result of sequencing errors. In an attempt to identify a targeted epitope, the translated sequence of each variant minigene was evaluated for predicted Class I MHC binding using the NetMHC Pred 3.4 tool [56] (available at http://www.cbs.dtu.dk/services/NetMHC-3.4/). Prediction was limited to MHC Class I H2^d^ molecules since mastocytoma P815 targets used in the screens do not express MHC class II. The peptide SYQKSINNI yielded strong predicted binding characteristics for H2-K^d^ and corresponded to a homologous, invariant sequence reported for *P*. *berghei* (PBANKA_1117700) MDH. This peptide was synthesized and tested by ELISPOT using frozen splenocytes from animals vaccinated with Vaccine 2 and from animals exposed to one or two doses of 1x10^4^ Py wild-type sporozoites with concurrent prophylactic azithromycin drug treatment to prevent onset of erythrocyte-stage infection. Splenocytes from all such mice responded to the PyMDH peptide, although the response in animals exposed to two doses of Py sporozoites was markedly reduced compared to a single dose of sporozoites (Fig 2). Sequencing of a genomic PCR product amplified from the *P*. *yoelii* 17XNL isolate used for PyRAS production confirmed that SYQKSINNI was the encoded peptide. Despite the aforementioned ambiguity in the Plasmodb database, sequencing revealed no evidence of non-synonymous variation within the SYQKINNI-coding region.

The same approach was used to bioinformatically predict H2^d^ and H2^b^ binders for PY00619, PY00638, and PY01906. However to date we have not identified any specific peptide epitopes from these proteins by ELISPOT screening (data not shown). Nonetheless, the PY00619, PY00638 and PY01906 minigene pools were indeed antigenic as they were each able to induce >100 SFU/million when administered to mice as a single pool gene gun immunization (data not shown). It is possible that some of the minigene-induced responses are directed at fusion peptides formed by the junction of the pathogen-derived peptide and the C-terminal LC3 fusion partner. Such antigens could not be present in the parasite itself and would therefore not be boosted upon challenge, however fusion peptides of this nature have not been formally tested. These data highlight the complexity of epitope prediction and suggest that the reactive peptide in the minigene screening stage was a different peptide than that predicted bioinformatically. For the purposes of this study, the peptides from PY00619, PY00638 and PY01906 were not pursued further.

**Fig 3:**
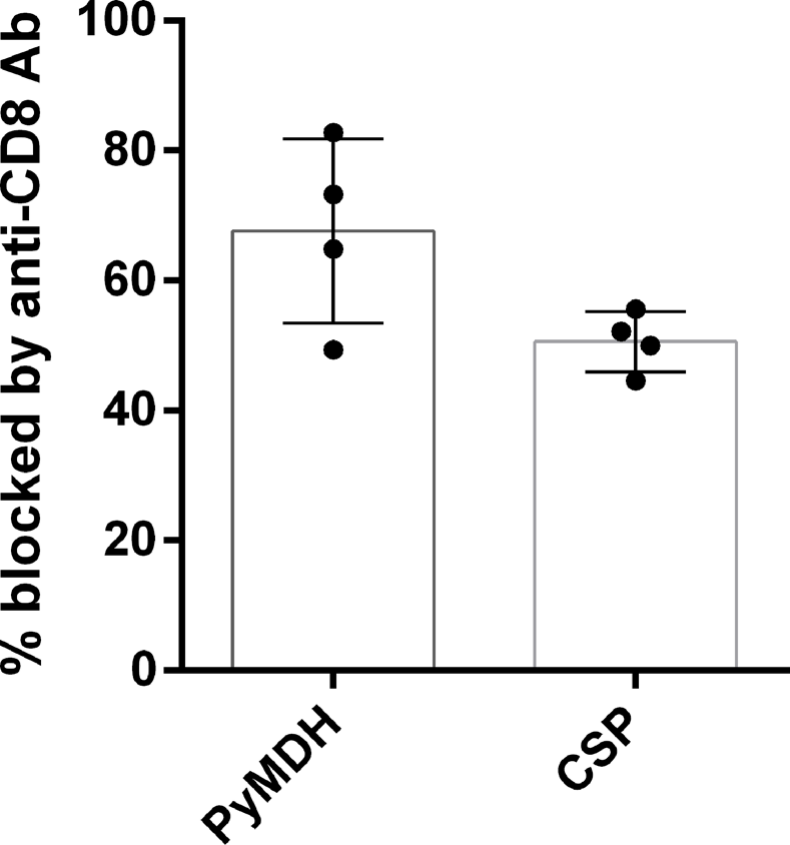
The PyMDH-specific response is CD8 T cell-dependent. ELISPOT wells were treated with anti-CD8 blocking antibody (clone 2.43, final 10 μg/mL) and compared to isotype-control treated wells. The y-axis shows the percentage reduction in SFU/million splenocytes for antibody-treated versus untreated wells. Splenocytes were from BALB/cj mice immunized with 5x10^4^ wild-type *P*. *yoelii* sporozoites administered with azithromycin prophylaxis on Days 0–2 post-challenge. Bars indicate mean and 95% confidence interval.

When ELISPOT assays were conducted in the presence or absence of anti-CD8a antibodies, PyCSP- and PyMDH-specific T cell responses were comparably reduced, indicating IFNγ production in both antigen-specific responses was CD8^+^ T cell-dependent (Fig 3).

### The MDH epitope SYQKINNI binds to murine H2-K^d^

**Fig 4:**
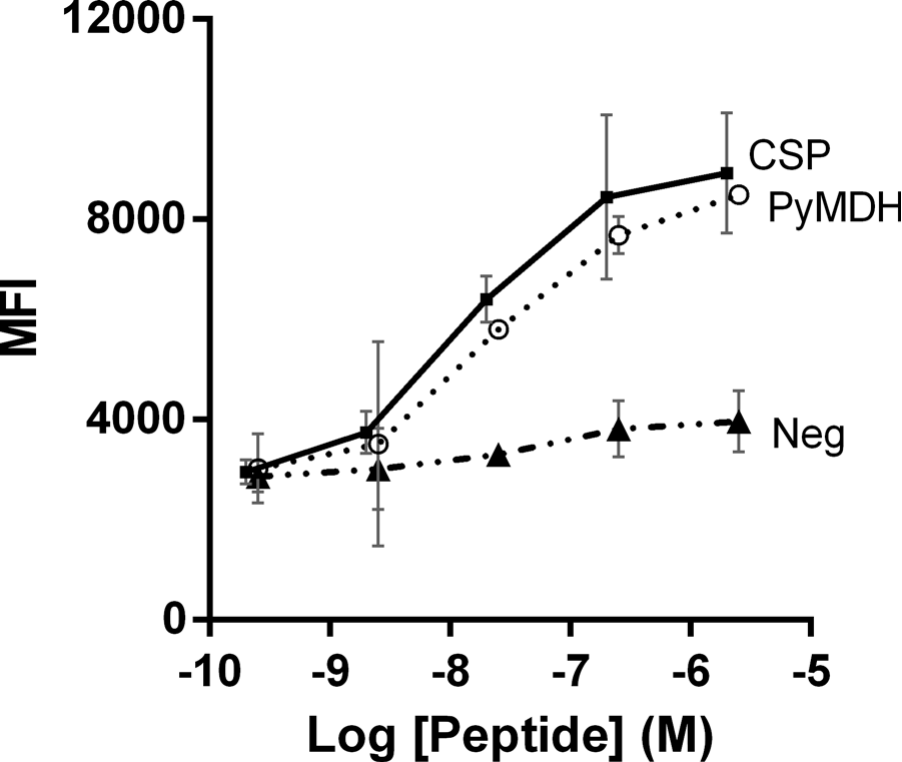
The PyMDH epitope binds to murine H2-K^d^. RMA/S cells expressing H2-K^d^ were incubated with peptides for PyCSP (squares), PyMDH (circles) or non-specific peptide. PyCSP and PyMDH peptides stabilized H2-K^d^, indicative of specific MHC binding. Error bars indicate the 95% confidence interval.

The affinity of SYQKSINNI for H2-K^d^ was determined using an RMA/S lymphoma cell line expressing H2-Kd as previously described [26, 54]. H2-K^d^ was stabilized by a known binder (PyCSP-derived SYVPSAEQI) and with similar affinity by PyMDH-derived SYQKSINNI. The apparent K_d_ for the peptides was 11.6 nM (PyMDH) compared to 9.6 nM (PyCSP) (Fig 4).

### PyMDH-specific CD8^+^ T cells are functionally cytotoxic

**Fig 5:**
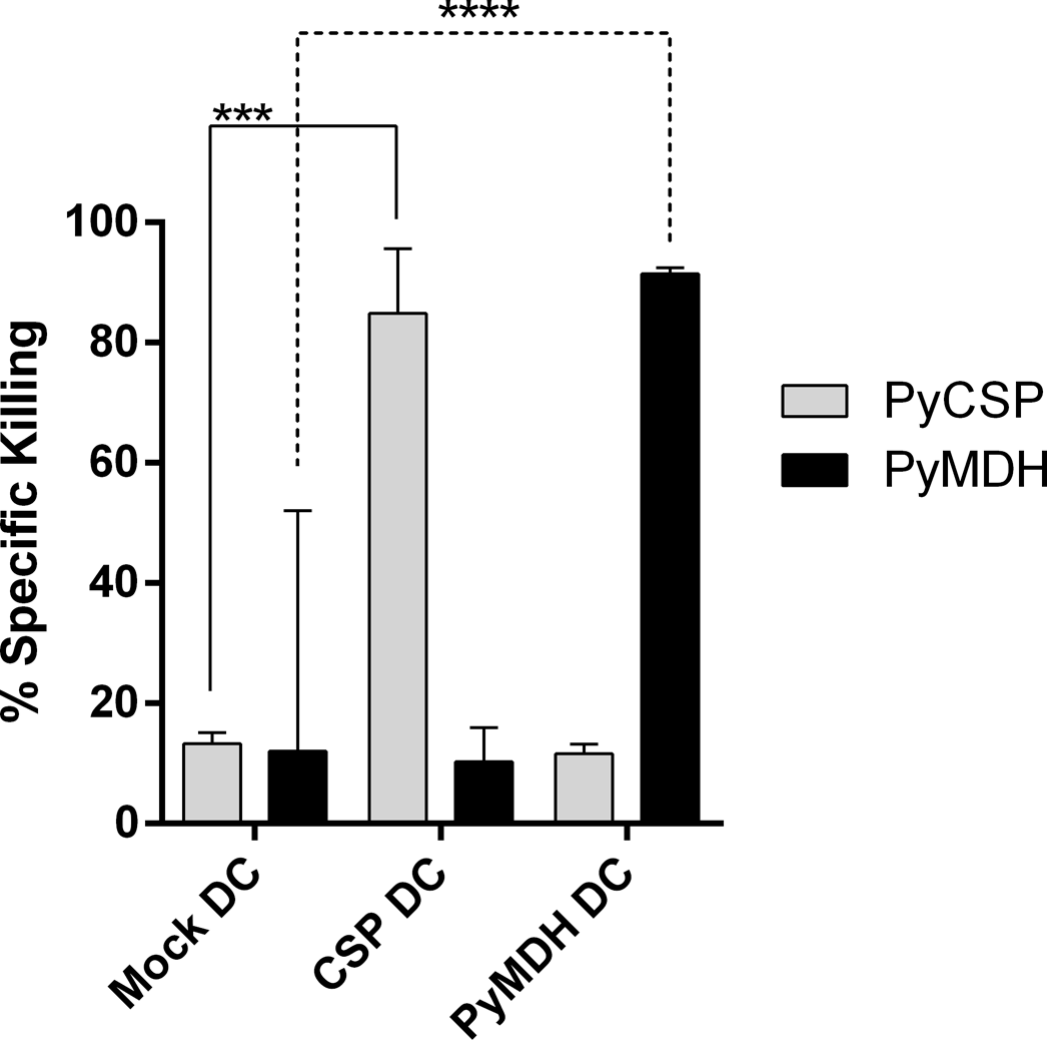
PyMDH-specific T cells are capable of antigen-specific cytotoxic killing. Mice were previously immunized with 1x10^6^ mature DCs pulsed with 1 μg/mL pf the antigenic peptide (PyCSP DC or PyMDH DC). Six days later mice were administered equal numbers of PyCSP peptide-coated CFSE^HI^ and PyMDH-peptide coated CFSE^LO^ target splenocytes; antigen-specific killing was monitored 18 hr later by flow cytometry. Like cytotoxic PyCSP-specific T cells, PyMDH-specific T cells demonstrate a high rate of antigen-specific killing. Bars indicate mean and 95% confidence interval; ***p<0.001; ****p<0.0001 (Student’s t test).

To evaluate the functional capacity of PyMDH-specific T cells, BALB/cj (Thy1.2^+^) mice were primed i.v. with 2x10^6^ GM-CSF-bone marrow-derived dendritic cells activated and pulsed overnight with lipopolysaccharide (0.1 μg/mL) and either 1 μg/mL PyCSP or PyMDH or no peptide. Six days later, splenocytes from Thy1.1 BALB/c mice were pulsed with PyCSP or PyMDH peptides or with no peptide and were differentially stained, mixed and injected into animals primed against individual peptides. Eighteen hr later, spleens were harvested and peptide-specific killing measured by flow cytometry. Animals immunized against the PyCSP peptide specifically killed 84% of PyCSP-pulsed targets, and animals sensitized to the PyMDH peptide killed 91% of PyMDH-pulsed targets (Fig 5).

### High-frequency PyMDH-specific CD8^+^ T cells do not protect mice from *P*. *yoelii* sporozoite challenge

**Fig 6:**
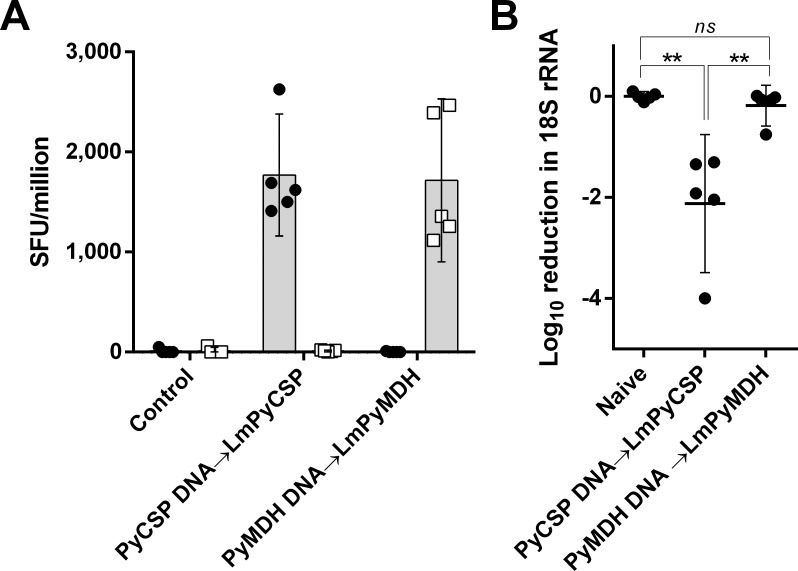
High frequency IFNγ-producing PyMDH-specific T cells do not protect against sporozoite challenge. BALB/cj mice were DNA gene gun vaccinated against Class I epitopes from PyCSP or PyMDH and boosted 21 d later with 5x10^6^ cfu attenuated *Listeria monocytogenes* expressing the same epitope. Mice were challenged with 1x10^4^ PyWT sporozoites i.v. and sacrificed for spleen and liver harvest 44 hr post-challenge. (A). ELISPOT performed on splenocytes at the 44 hr time point reflects the frequency of antigen-specific T cells at the time of challenge. Circles, PyCSP peptide; squares, PyMDH peptide; bars indicate mean and 95% confidence interval. (B). Liver stage infection was monitored by *Plasmodium* 18S rRNA RT-PCR. Bars indicate mean and 95% CI for *Plasmodium* 18S rRNA normalized to mouse GAPDH mRNA content; **p < 0.01 (Student’s t-test).

Five animals per group were vaccinated with either PyCSP or PyMDH using a DNA prime/*Listeria* boost protocol. At a memory time point, all vaccinated mice plus five naïve infectivity control mice were challenged with 1x10^4^ wild-type *P*. *yoelii* sporozoites. ELISPOT performed at the time of liver harvest demonstrated antigen-specific, high frequency IFNγ-producing responses to PyCSP and PyMDH in appropriately immunized mice (Fig 6A). Compared to infectivity control mice, 5/5 PyCSP-vaccinated mice showed significant vaccine-induced protection, with one animal showing undetectable Py 18S RNA in the liver indicating sterile protection (Fig 6B). In contrast, the Py 18S rRNA concentration in animals vaccinated with PyMDH was indistinguishable from that of the infectivity controls (Fig 6B), indicating no protection despite high frequency PyMDH-specific T cell responses.

### Heterologous cross-species immunization with a late-arresting sporozoite re-expands the PyMDH-specific T cell population more than homologous immunizations

**Fig 7:**
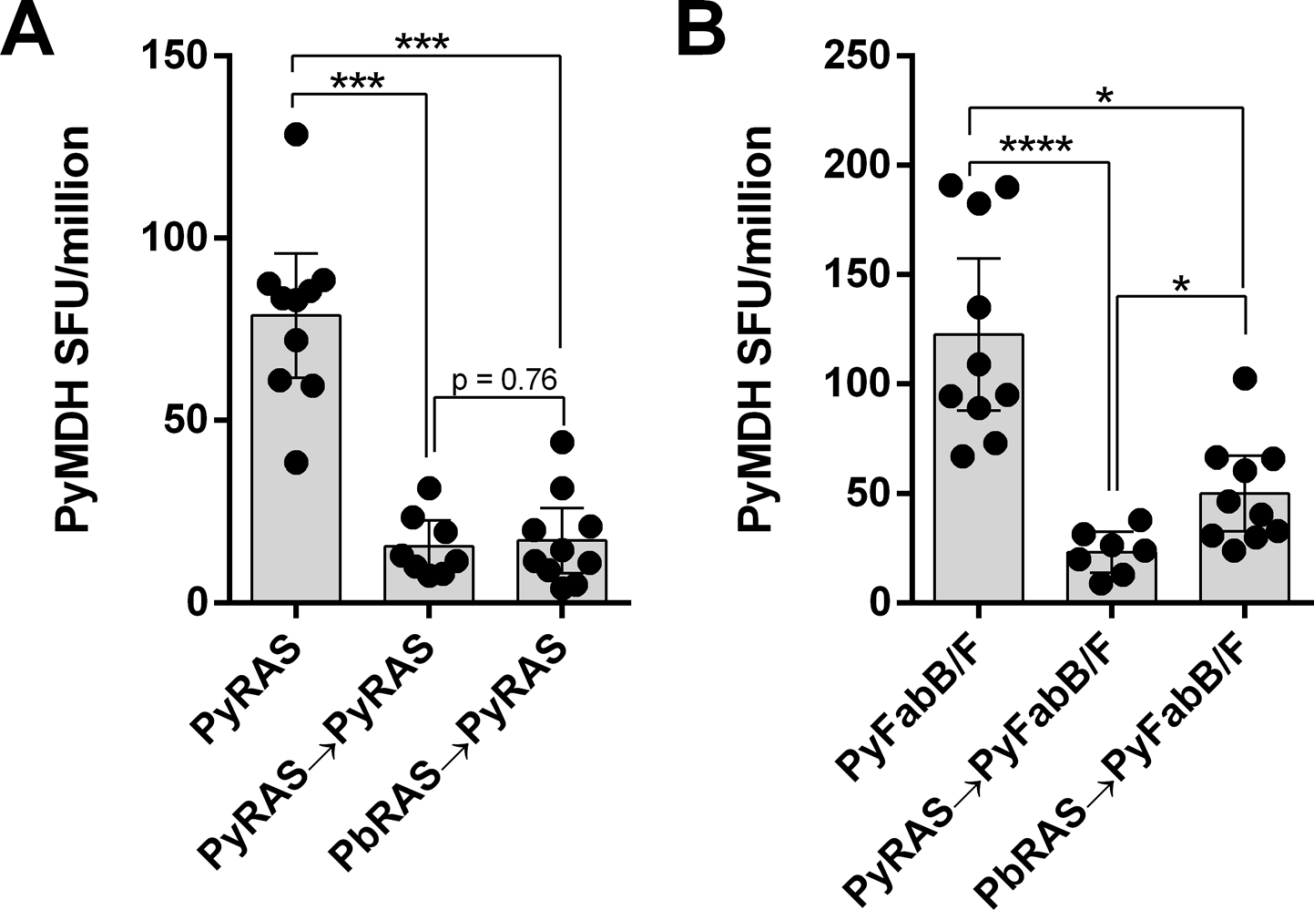
PyMDH-specific T cells are not recalled with repeated homologous sporozoite immunization but can be recalled by heterologous cross-species immunization. (A) BALB/cj mice were immunized with nothing or with 1x10^4^ PyRAS or PbRAS and three wks later all groups received 1.5x10^5^ purified PyRAS. IFNγ ELISPOT was performed on splenocytes obtained 6 days after the final vaccination. (B) BALB/cj mice were immunized with nothing or with 1x10^4^ PyRAS or PbRAS and three wks later all groups received 1.5x10^5^ purified, genetically-attenuated Pyfabb/f- sporozoites. IFNγ ELISPOT was performed as in (A). Bars show mean +/- 95% confidence interval; * p<0.05, ***p<0.001; ****p<0.0001 (Student’s t-test).

Previous studies showed that T cell responses to pre-erythrocytic antigens could either be expanded by repeated homologous (same-species) sporozoite immunization (e.g., CSP-specific CD8 T cells) or failed to re-expand (e.g., L3-specific CD8 T cells) [26]. Recent unpublished work has shown that immunization with two different murine-infecting *Plasmodium* species (*P*. *yoelii* 17XNL and *P*. *berghei* ANKA) could recall the L3-specific response to the epitope shared between species if the secondary immunization was with a late-arresting, genetically-attenuated sporozoite (S. Murphy, pers. comm.–manuscript in review). Since the PyMDH epitope was also conserved between *P*. *yoelii* and *P*. *berghei*, mice underwent homologous or heterologous immunizations with combinations of PyRAS, PbRAS and Pyfabb/f- parasites. As observed for L3, when RAS parasites were used, neither homologous (PyRAS→PyRAS) nor heterologous (PbRAS→PyRAS) regimens re-expanded the PyMDH-specific cells (Fig 7A). However, when the secondary immunization was switched to the less-attenuated Pyfabb/f- strain, the heterologous regimen (PbRAS→Pyfabb/f-) expanded PyMDH-specific responses more so than the homologous regimen (PyRAS→Pyfabb/f-), although not to the level observed with a single Pyfabb/f- immunization (Fig 7B). This finding suggests that PyMDH-specific responses and those against other proteins with similar protein expression kinetics may be boosted by heterologous more so than homologous species immunizations.

## Discussion

Development of antibody-based subunit vaccines has been aided by the inherent stability, sensitivity and soluble nature of antibodies in serum. In contrast, the development of T cell-based vaccines requires evaluation of live cellular MHC-restricted interactions between T cells and MHC-matched APCs displaying the cognate target antigen. For intracellular pathogens expressing thousands of potential targets such as malaria, this requirement has inhibited discovery of protective vaccine subunits. The synthetic minigene vaccine technology described here is designed to enable the rapid evaluation of T-cell responses against large numbers of pathogen-derived proteins, in order to identify both dominant and sub-dominant antigens.

In these proof-of-principle studies, minigene vaccination resulted in the identification of four novel T cell antigens from among 89 pre-erythrocytic stage *P*. *yoelii* proteins. These included one dominant antigen encoded by PY03376 (PyMDH), and three antigens encoded by PY00619, PY0638 and PY01906 minigene pools. We identified the peptide SYQKSINNI as a major PyMDH epitope. Robust responses against PyMDH were detected in animals receiving the vaccine alone and in response to sporozoite immunization in the absence of DNA vaccination. PyMDH-specific responses in DNA-vaccinated animals could be recalled by sporozoites. However, despite recent reports of a protective T cell response against *Toxoplasma gondii* malate dehydrogenase [57], no protection against PyMDH was observed following DNA prime/*Listeria* boost vaccination despite induction of extremely high frequency, cytotoxic PyMDH-specific CD8^+^ T cell responses. The PyMDH response was also evaluated in mice exposed to one or two rounds of sporozoite immunizations. The PyMDH-specific response was not potently recalled by homologous sporozoite immunizations, although the epitope is shared with *P*. *berghei* ANKA MDH and recall could be partially augmented by immunization with two different species of murine-infecting *Plasmodium* sporozoites. Thus, PyMDH appears to be an antigen in the same category as that of the recently described pre-erythrocytic *P*. *yoelii* ribosomal protein L3, which also displayed robust pre-erythrocytic immunogenicity but no protective efficacy and poor recall by repeated homologous sporozoite exposures [26].

The other antigenic minigene pools have not been characterized to the same extent as PyMDH. Animals receiving PyRAS 16 days after the final DNA library vaccine exhibited low level responses against PY00619 and PY00638 minigene pools but not to PY01906, and additional studies will be required to determine whether these responses represent true parasite recall or simply residual, declining responses from the initial DNA vaccination. Each of these three minigene pools was able to induce IFNγ responses in mice vaccinated with single pool DNA vaccines. The antigenic epitope(s) for PY00619, PY00638 and PY01906 are currently unknown and additional studies are ongoing to evaluate these antigens further.

The fact that the robust responses against PyMDH are not protective suggests that the epitope may not be displayed on the surface of an infected hepatocyte harboring live proliferating parasites, or may be presented by the hepatocyte at a time point that is too late to provide protection. To be displayed on the surface of the hepatocyte, PyMDH would probably need to be exported from the parasite and across the hepatocyte-derived vacuole membrane to the hepatocyte cytoplasm. However unlike erythrocyte-stage parasites [47, 48], there is currently no known consensus motif that directs pre-erythrocytic protein export from the vacuolar parasite to the hepatocyte cytoplasm. Responses to PyMDH-like epitopes may be driven either entirely or in part by cross-presentation of dead or dying parasites. We [26] and others [58, 59] have identified other antigens with similar response profiles. This type of antigen may represent an immune evasion strategy employed by the parasite whereby epitopes derived from proteins that are not protective serve as “decoys”, directing immune system resources toward non-protective targets. There is increasing evidence that this decoy antigen phenomena also occurs in bacterial and viral infections [60, 61]. Such antigens may be under selective pressure to remain invariant as T cell responses against them could promote survival of the parasite. In contrast, T cell epitopes from proteins that are protective are likely to be expressed and presented by hepatocytes during liver-stage infection and therefore may be under selective pressure to alter their primary sequence to avoid immune detection. Separating antigenic protective antigens (the “wheat”) from dispensable antigens such as PyMDH and *P*. *yoelii* ribosomal protein L3 (the “chaff”) represents a major hurdle to our understanding of the *Plasmodium*-specific T cell repertoire and to development of effective subunit vaccines for malaria.

Both multi-gene DNA library vaccines elicited responses against just two novel antigens each. Thus, a major question concerning our ‘highly parallel immunization’ strategy is whether immunization with highly complex DNA vaccines is capable of stimulating responses against a highly diverse set of targets, even when small subsets of minigenes are segregated and delivered to different APCs via different gold bead carriers. The malaria literature indicates that mixtures of small numbers of *Plasmodium* genes encoding *bona fide* antigens are immunogenic in mice and non-human primates [41, 42] and humans [43], although the quantity of DNA and the administration methods differ from the gene gun approach reported here. In one mouse study, administration of a mixture of five *P*. *falciparum* antigen-coding plasmids led to alterations (CSP and Exp1 decrease and LSA1 increase) in T cell immunogenicity compared to immunization with single gene plasmids only [62]. During construction of the vaccines reported here, each of the more than 100 minigene pools (10 cloned minigenes/pool) in each vaccine were individually precipitated onto separate aliquots of gold beads in parallel in an effort to promote diverse responses against multiple vaccine-encoded antigens. Such physical segregation of antigens onto different pools of gold beads is known to promote both antibody and T cell response diversity in other studies of gene gun-delivered antigens [44–46]. However, the minigene library vaccine represents a much larger set of antigens than studied in the aforementioned references, and we are uncertain how the degree of diversity and the absolute quantity of any individual antigen-encoding DNA ultimately affect immune response outcomes. In addition, a small portion of some proteins were not included in the library because correct sequences were not recovered following dial-out error correction. Therefore, some bona fide antigens could also have been encoded in the omitted sequences. Given these caveats, the library vaccination-screening strategy described herein can *rule in* T cell responses, but is not sufficiently sensitive in its current form to *rule out* immunogenicity or protective effects of targets where responses are not detected or for peptides not covered for specific proteins.

Given the suboptimal performance of the CSP control, we are currently exploring several methods to increase the intensity and breadth of responses induced by complex DNA library vaccines. First, as mentioned earlier, we have adopted a standard 5’ UTR and a ubiquitin fusion design that have proven superior to the design used in the LC3-based vaccines. Ubiquitin-tagged proteins have shown increased CD8^+^ T cell immunogenicity in many systems [63–67] including for some malaria DNA vaccine antigens [68]. Simply increasing the dose of DNA on gold beads is unlikely to yield a significant effect as previous titration studies with the gene gun [52] suggest that the dose of DNA required to stimulate a response is well within the range of each construct achieved in this protocol. In contrast, increasing the number of DNA vaccination cartridges administered per animal would result in an increase in the number of DNA-coated particles delivered to dermal APCs, potentially boosting the number of responders induced against multiple antigens. In addition, using the sub-optimal CSP minigene, we have tested novel adjuvants targeting B7 family inhibitory receptors and have observed a significant increase in the number of responders induced (B. Stone, S. Murphy, unpublished observation). Furthermore, we tested a P815 cell line that overexpresses the co-stimulatory molecule B7.1 [69] as APCs in minigene-transfected ELISPOT screening assays and observed five-fold higher sensitivity for IFNγ producing cells, indicating that additional antigens may be discoverable by ELISPOT with optimized transfection-compatible APCs. Vaccination studies testing these improvements using these and additional minigene libraries are currently underway.

## Supporting Information

S1 Table Minigene library vaccine minigenes. Microsoft Excel formatted spreadsheet with two worksheets (Vaccine 1 and Vaccine 2) listing the Vaccine (column A), gene ID (column B), product description (column C), minigene name (column D), vaccine-specific pool number (column E), minigene sequence (column F), encoded peptide (column G), common and pool-specific primers (columns H-I) and percentage coverage for each protein in the final error-corrected library (column J).
(XLSX)
